# Cystogastrostomy as an alternative treatment for recurrent huge infected hepatic cyst

**DOI:** 10.1055/a-2329-2093

**Published:** 2024-06-07

**Authors:** Hau-Jyun Su, Ting-An Lin, Ssu-Yu Chen, Ming-Chang Tsai, Edy Kornelius, Yu-Ting Lin, Chi-Chih Wang

**Affiliations:** 1 63276 Division of Gastroenterology and Hepatology, Department of Internal Medicine, Chung Shan Medical University Hospital, Taichung, Taiwan; 2 34899 School of Medicine, Chung Shan Medical University, Taichung, Taiwan; 3 Institute of Neuroscience, National Yang-Ming Chiao Tung University, Taipei, Taiwan; 4 34899 Institute of Medicine, Chung Shan Medical University, Taichung, Taiwan; 5 63276 Department of Internal Medicine, Chung Shan Medical University Hospital, Taichung, Taiwan; 6 63276 Department of Anesthesia, Chung Shan Medical University Hospital, Taichung, Taiwan

A 73-year-old woman with a history of polycystic liver and kidney disease (PLKD) presented with coffee grounds emesis, hypotension, and acute on chronic renal failure. She developed septic shock and a decline in renal function, necessitating emergency hemodialysis after admission. She had undergone robotic fenestration of a large hepatic cyst 5 months earlier because of acute cholangitis resulting from compression of the common bile duct by the cyst. Esophagogastroduodenoscopy revealed duodenal ulcer and lumen narrowing at the second portion of the duodenum ( Fig. 1 **a** ). Magnetic resonance cholangiopancreatography confirmed a recurrent huge hepatic cyst at segment IV of the liver with air-fluid level ( Fig. 1 **b** ).

**Fig. 1:**
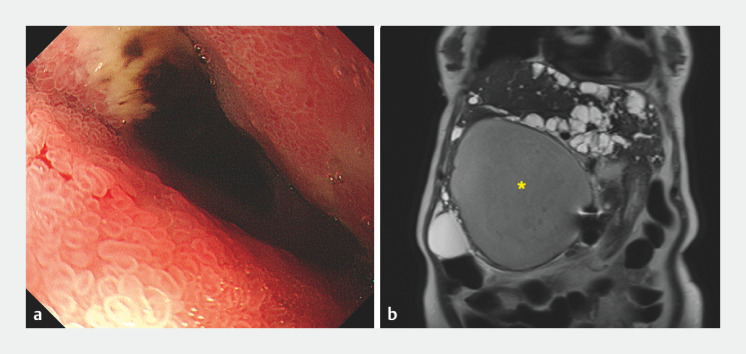
Initial investigations. **a** Esophagogastroduodenoscopy revealed duodenal obstruction: duodenal ulcers with lumen narrowing at the second duodenal portion. **b** Magnetic resonance imaging showed a huge hepatic cyst (asterisk), 17 cm in diameter, deriving from segment IV of the liver, causing duodenum compression. The presence of numerous cysts of the liver and kidneys is suggestive of polycystic liver and kidney disease.

She underwent endoscopic ultrasound (EUS)-guided transmural drainage using an Olympus UCT-260 echoendoscope (Olympus, Tokyo, Japan) ( Video 1 ). The cyst was first punctured from the stomach by a 19-gauge fine needle ( Fig. 2 ). Subsequently, a guidewire was advanced into the cyst, followed by dilation using an ES Dilator (Zeon Medical Co., Tokyo, Japan) and 4-mm Cook Titan balloon catheter (Cook Medical, Bloomington, Indiana, USA). Finally, two double-pigtail plastic stents were placed to create a cystogastrostomy ( Fig. 3 ). Following drainage and antibiotic treatment, the patient’s renal function and sepsis gradually improved, and she was discharged without requiring hemodialysis.

**Fig. 2:**
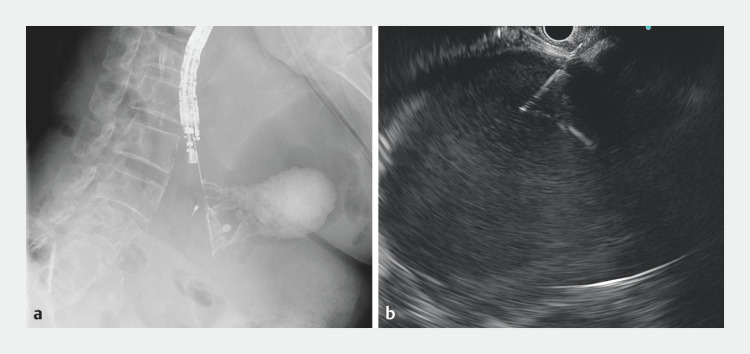
The hepatic cyst was punctured from the stomach. **a** Endoscopic ultrasound (EUS) with contrast injection under fluoroscopy. **b** EUS view.

**Fig. 3:**
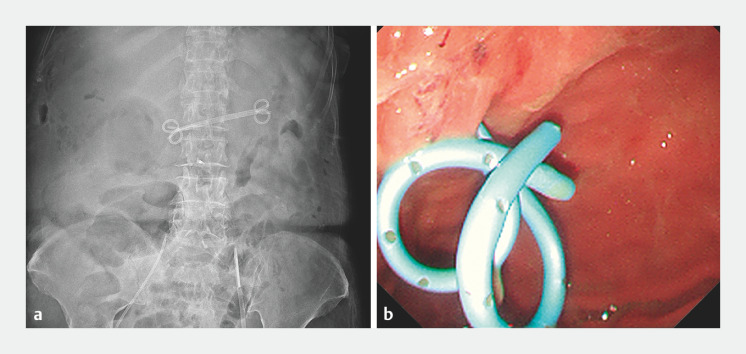
Two double-pigtail plastic stents were placed between the cyst and the stomach, with drainage of pus into the stomach. **a** Abdominal radiography. **b** Endoscopic view.

Endoscopic ultrasound-guided cystogastrostomy for a huge infected hepatic cyst.Video 1

Sonography 1 month later showed a decrease in cyst size with residual debris inside ( Fig. 4 ), and the patient remained asymptomatic.

**Fig. 4:**
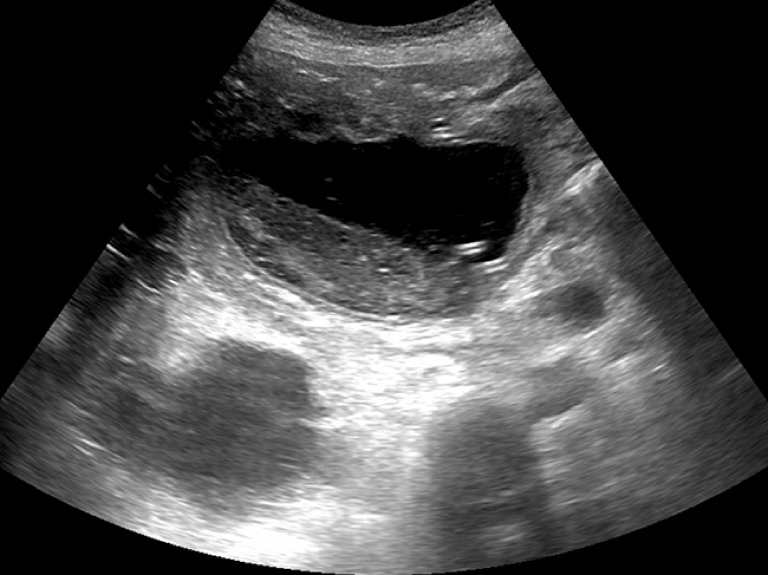
Sonography 1 month later showed a decrease in cyst size, with residual debris inside.

Although symptomatic hepatic cyst can be effectively managed by laparoscopic fenestration, the recurrence rate remains high in patients with PLKD 1 . Endoscopic cystogastrostomy is initially used for pancreatic pseudocysts 2 or walled-off necrosis 3 . Some reports have shown that EUS-guided transmural drainage could be used effectively to manage hepatic abscesses or infected hepatic cysts 4 5 . This case report presents the first instance of treating a recurrent infected hepatic cyst by EUS-guided double-pigtail plastic stents as primary drainage in a patient with PLKD. We propose it as a rescue option for recurrent infected hepatic cysts when the patient is not a candidate for surgery.

Endoscopy_UCTN_Code_TTT_1AR_2AK

## Notes

Endoscopy E-Videos https://eref.thieme.de/e-videos
*E-Videos* is an open access online section of the journal *Endoscopy* , reporting on interesting cases and new techniques in gastroenterological endoscopy. All papers include a high-quality video and are published with a Creative Commons CC-BY license. Endoscopy E-Videos qualify for HINARI discounts and waivers and eligibility is automatically checked during the submission process. We grant 100% waivers to articles whose corresponding authors are based in Group A countries and 50% waivers to those who are based in Group B countries as classified by Research4Life (see: https://www.research4life.org/access/eligibility/ ). This section has its own submission website at https://mc.manuscriptcentral.com/e-videos .
